# High Prevalence of *GALC* Gene Variants in Adults With Neurodegenerative Conditions

**DOI:** 10.1111/ene.70206

**Published:** 2025-05-20

**Authors:** Federica Feo, Luciana Tramacere, Silvia Ramat, Alessandra Govoni, Luca Caremani, Giulia Grigioni, Davide Mei, Silvia Falliano, Francesca Marin, Lorenzo Ferri, Antonella Paoli, Marina Rinaldi, Giancarlo la Marca, Daniela Ombrone, Elena Procopio, Renzo Guerrini, Amelia Morrone, Anna Caciotti

**Affiliations:** ^1^ Department of Neuroscience, Pharmacology and Child Health University of Florence Florence Italy; ^2^ Neuroscience and Medical Genetics Department Meyer Children's Hospital IRCCS Florence Italy; ^3^ USL Toscana Centro, Neurology Unit Ospedale San Giovanni di Dio Florence Italy; ^4^ Parkinson Unit AOU Careggi Florence Italy; ^5^ Department of Experimental and Clinical Biomedical Sciences University of Florence Italy

**Keywords:** GALC, Krabbe disease, leukoencephalopathy, parkinsonism, risk factors

## Abstract

**Background and Purpose:**

Galactocerebrosidase (GALC) deficiency causes Krabbe disease, a severe lysosomal neurodegenerative condition. Emerging evidence suggests that heterozygous *GALC* variants may contribute to multiple sclerosis, attention‐deficit hyperactivity disorder, and synucleinopathies. We aim to investigate the potential association between *GALC* heterozygous variants and neurodegenerative disorders, expanding on existing literature.

**Methods:**

We screened 110 adults with symptoms shared by lysosomal storage disorders (LSDs) and common neurodegenerative diseases, such as Parkinson's disease, Lewy body dementia, and ataxias of different etiology.

**Results:**

We found *GALC* heterozygosity in this group to be notably enriched, approximately 1 in 28, compared to 1 in 150 in the general population. This led to a focus on 11 individuals with pathogenetic *GALC* variants and/or the disease‐associated polymorphism p.(Arg184Cys). One patient, compound heterozygous for a pathogenetic variant and the p.(Arg184Cys), exhibited reduced  GALC activity and a clinical course consistent with late‐onset Krabbe disease. In another patient, we found the very rare synonymous variant p.(Leu238Leu) in the *GALC* gene. Two patients carrying known pathogenetic *GALC* variants were also heterozygous for other known pathogenetic variants in other LSD‐associated genes, including *HEXB* (Sandhoff disease) and *GUSB* (mucopolysaccharidosis VI).

All the 11 patients in the selected cohort exhibited symptoms similar to atypical Parkinson's disease and a high frequency of leukoencephalopathy, inflammatory disorders, and cancer.

**Conclusions:**

Our findings indicate a possible connection between the patients' neurodegenerative conditions and *GALC* defects, including disease‐associated polymorphisms and silent variants. Additional genetic alterations affecting sphingolipid and glycosaminoglycan metabolism may act as contributing factors.

## Graphical abstract

1. *GALC* Defects and Neurodegenerative Impact:
  Exploring the role of GALC variants in adults with neurodegenerative symptoms and identifying an undiagnosed case of adult‐onset Krabbe disease.
2. Metabolic Interplay:
  Focus on interrelated pathways as potential risk factors for neurodegeneration in heterozygous lysosomal storage disorder carriers.

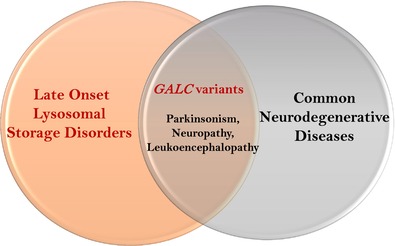

## 1 Introduction

The *GALC* gene encodes galactoctosylceramidase or galactocerebrosidase (GALC), an enzyme that degrades sphingolipids such as galactosylceramide (Gal‐Cer), psychosine, and lactosylceramide by hydrolyzing galactose ester bonds [1]. The altered activity of GALC leads to defective myelination through two putative pathogenetic mechanisms. The first hypothesizes an altered lipid degradation during the myelin turnover, whereas the second hypothesizes a toxic accumulation of psychosine in the myelin‐forming cells, specifically oligodendrocytes and Schwann cells [1, 2]. The activity of GALC on Gal‐Cer is required in the remyelination pathway during myelin turnover [2]. However, abnormal multinucleated globoid cells, resulting from macrophages activated by free Gal‐Cer in the brain of patients, are the only known direct link between Gal‐Cer and altered neuronal metabolism [3]. In addition to degrading its primary substrate, GM1 ganglioside, the beta‐galactosidase enzyme can also act on Gal‐Cer [4, 5], which could explain why Gal‐Cer does not accumulate in the nervous system of patients with Krabbe disease (KD) [1, 2].

The pathological features of the nervous system in patients with KD are largely caused by psychosine accumulation [6]. Psychosine, a basic lipid of oligodendrocytes and Schwann cells, is a potent neurotoxin at nonbasal concentrations [7]. Its accumulation may lead to disorganization of myelin compounds, axonal defects, inflammation, and α‐synuclein fibrillization likewise observed in Parkinson's disease (PD) [6, 7]. Inflammatory events in KD can also be caused by myelin/cell debris unrelated to psychosine [8]. It is suggested that psychosine directly promotes α‐synuclein aggregation by direct physical interactions [9]. All of these molecular mechanisms affect multiple cellular functions, leading to demyelination and neuroinflammation, resulting in the severe neurodegeneration and leukodystrophy of KD.

The globoid cell leukodystrophy described by Krabbe in 1916 is estimated to occur in 1/100,000 to 1/250,000 live births [10]. Depending on symptoms onset, the disease is classified as early infantile, late infantile, juvenile, or adult forms [11]. Cognitive and motor deterioration, loss of vision, and sensory motor neuropathy are the main symptoms of the disease; later onset forms progress more slowly and cause less severe manifestations [11]. Adult forms usually cause unsteady gait with frequent falls/cerebellar ataxia, weakness, and peripheral neuropathy [12]. The early‐onset form of the disease accounts for approximately 90% of Krabbe patients [13, 14], though approximately 17% of Krabbe patients reported in the literature could be recategorized as belonging to the adult subgroup [10].

Sphingolipid catabolism is involved in many mono‐ or multiorgan pathologies, which are associated with neurodegeneration and a high mortality rate [15]. Associations have been described between GALC and neurodegeneration in synucleinopathies and PD [16, 17], multiple sclerosis (MS) [18], and attention‐deficit hyperactivity disorder (ADHD) [19].

In PD, variants of the *GBA* gene, mutated in Gaucher disease and encoding beta‐glucosidase, are well‐established risk factors. Emerging evidence suggests that numerous other genes associated with lysosomal storage disorders (LSDs) may also contribute to increased PD susceptibility. These include *SMPD1* (Niemann–Pick A), *NEU1* (sialidosis), *NAGLU* (Sanfilippo B), *GUSB* (mucopolysaccharidosis VII), *GRN*, which dominantly causes frontotemporal dementia, and *GALC* [20].

Recent studies indicate that a genetic risk score, derived from a complex polygenic background, can influence the penetrance of mutations in well‐established genetic modifiers, such as those in *GBA* [21]. Furthermore, variants in *GRN* and *ARSA* (linked to metachromatic leukodystrophy) have been implicated in modulating the dementia phenotype in individuals from families affected by Alzheimer's disease [22]. In adults, neurodegenerative conditions of unclear etiology may present with symptoms commonly observed in LSDs, including ataxia, myoclonus, dementia, and psychiatric manifestations [23].

In order to explore potential associations between LSDs defects and neurodegeneration, using a panel of genes known to cause late‐onset LSDs, we performed NGS in a series of adult patients with atypical PD, parkinsonism, Alzheimer's disease, or ataxia.

Our findings show a high prevalence of *GALC* variants, supporting their neurodegenerative role beyond lysosomal pathology.

## 2 Methods and Materials

### 2.1 Criteria for Patients' Inclusion

The study included patients of Italian origin. Patients were included if they had at least one major neurological symptom including ataxia, cognitive impairment, parkinsonism with the following red flags: early falls, rapidly progressive dementia, and early perceptual disturbances, dysarthria, epilepsy, myoclonus, tremor, neuropathy, and behavioral problems such as hyperactivity, and two or more minor non‐neurological manifestations such as coarse facies, hearing and visual loss, organomegaly, multiplex dysostosis, cardiomyopathy, lymphomas, and polyclonal gammopathy. Exclusion criteria were brain injury or tumors, stroke, postinfectious or prenatal brain abnormalities, and known genetic conditions.

### 2.2 Sequencing

We studied 64 genes associated with lysosomal storage disorders by next‐generation sequencing (NGS) using a custom‐designed panel (Illumina, San Diego, CA) (detailed in Table SS1). Libraries using the Nextera Rapid Capture Enrichment kit (Illumina) according to the manual instructions were prepared. We annotated variants both with the BaseSpace Variant Interpreter platform of Illumina (https://platform.login.illumina.com/) and by the Franklin by Genoox platform (https://franklin.genoox.com/clinical‐db/home). Analogously, exome analyses of Pt1 and his relatives were performed by a paired end 150 bp protocol proceeded on the NextSeq 500 platform (Illumina).

The CoNVaDING tool (Copy Number Variation Detection In Next‐generation sequencing Gene panels) by Johansson et al. (2016) was used to detect gross rearrangements in the 64 genes panel. Sanger sequencing was performed to confirm the retained variants in the probands and subsequently to evaluate variants segregation by analyzing parents' DNA, if available.

### 2.3 RNA Quantitation

*GALC* mRNA quantitation of lymphocyte samples was performed as previously described [24]. We quantified and normalized *GALC* exons to the endogenous reference *18S* rRNA gene (Pre‐Developed TaqMan assay reagents, ref. 4333760T, Applied Biosystems, UK) using TaqMan assays. We performed splicing predictions by Alamut software (http://www.interactive‐biosoftware.com/alamut.html), based on five different algorithms (SpliceSiteFinder, MaxEntScan, NNSPLICE, GeneSplicer, Human Splicing Finder), to predict the effect of the c.714C>G, p.Leu238Leu genetic variation on the *GALC* mRNA splicing.

### 2.4 Enzyme Assays by LC–MS/MS

Dried blood spot (DBS) samples were prepared to screen GALC enzyme activity using the NeoLSD MSMS kit (PerkinElmer, Turku, Finland) as described elsewhere [25].

We measured the samples by Flow Injection Mass Spectrometry system using multiple reaction monitoring with an API3200 instrument (Sciex, Toronto, Canada). Lysosomal enzymatic activities were expressed as μmol/L/h.

Individuals who exhibited GALC activity levels below 0.7 μmol/h/L were deemed biochemically positive for KD.

### 2.5 Enzyme Assays by Spectrofluorometric Analysis

The BCA protein assay macro kit (Serva, ref. 832.39228/02) was used to set up the starting proteins used in each enzyme assay. GALC activity in cell lysates of leukocytes was measured as previously described [26].

### 2.6 *In Silico* Structural Analyses

We analyzed single amino acid substitutions in the GALC protein using the dbNSFP database (https://sites.google.com/site/jpopgen/dbNSFP). GALC protein modeling, prediction, and analysis were performed using two bioinformatics tools. We designed the structure by submitting the entire sequence that comprises 41–668 residues on Phyre2 software (http://www.sbg.bio.ic.ac.uk/phyre2/html/page.cgi?id=index). We achieved the molecular graphic of the wild‐type GALC enzyme with UCSF ChimeraX (https://www.cgl.ucsf.edu/chimerax/) [27]. Protein–protein interaction and functional enrichment analysis using STRING (https://string‐db.org/) and GeneMANIA (https://genemania.org/) helped to predict the GALC networks and sets. To select neurodegenerative conditions and possible causes and risk factors, we consulted the Ontology browser (https://hpo.jax.org/app/browse/term/).

### 2.7 Statistical Analysis

The GraphPad Prism Software version 5.02 was used for statistical analysis. We expressed data from enzyme assays as the median, maximum, and minimum values in a box plot graph. Differences between groups were determined using unpaired two‐tailed Student's *t* test in order to determine statistical significance (set at *p* < 0.05 vs. control).

### 2.8 Ethical Standards

We conducted this study in accordance with the ethical standards of the institutional research committee and with the 1964 Helsinki Declaration and its later amendments. It was also approved by the ethics board review of the Tuscany Region, “Health Research Call 2018” for the study “LYSOLATE” (Ref CEAVC 16774, approved on May 5, 2020). According to ethical guidelines, all blood samples for storage and analysis were obtained only after patients (and/or their family members') gave written informed consent. The patients' informed consent contained permission to share scientific results.

## 3 Results

### 3.1 Molecular Analyses

For NGS analysis, we used a panel of 64 genes associated with LSDs in 110 unrelated patients with neurodegenerative diseases.

Here, we specifically describe the results obtained in 11 patients whose NGS analysis revealed significant variants in the *GALC* gene. These variants include known pathogenetic variants, the c.550C>T p.(Arg184Cys) disease‐associated polymorphism, and the very rare c.714C>G, p.(Leu238Leu) synonymous variant (see Table 1).

**TABLE 1:** Clinical and genetic features of the 11 selected patients with *GALC* variants.

Patient	Pt1	Pt2	Pt3	Pt4	Pt5	Pt6	Pt7	Pt8	Pt9	Pt10	Pt11
Sex	m	f	f	m	f	m	m	f	f	m	f
*GALC* genotype^a^	[p.I384T] + [p.R184C]	[p.Y370S]	[c.1034‐2A>G]	[p.Y319C]	[p.R184C]	[p.R184C]	[p.R184C]	[p.R184C]	[p.R184C]	[p.R184C]	[p.L238L]
Onset (years)	2	14	54	67	58	60	53	35	56	70	19
Neurological features	Dementia, dysarthria, unsteady gait/ataxia, epilepsy	Peripheral neuropathy, vertigo	A‐PD, peripheral neuropathy	A‐PD, cognitive decline	PD	A‐PD, peripheral neuropathy myoclonus	Tremor, peripheral neuropathy	PD, dysphagia	Cerebral ischemia	Cognitive decline, A‐PD, REM sleep disorder, nystagmus	Peripheral neuropathy, tremor
Psychiatric disorders	Yes	No	No	No	Yes	No	Yes	Yes	Yes	Yes	No
Hearing loss	Yes	Yes	Yes	Yes, after head trauma	No	No	No	No	No	No	No
Impaired vision	Blindness	Hypovirus	Cataract	No	No	Cataract	No	No	Retinopathy	No	No
Cardiac pathology	No	No	No	No	No	Arrhythmia	3 IMAs	No	No	Ischemia	No
Immunological features	No	IgA deficit	No	Asthma	Monoclonal gamma peak	Connectivities	No	Asthma	EGPA	No	No
Oncological history	No	TC, NHL, MM	Breast cancer	No	Lung cancer	TC, vocal cord carcinoma	HL	MM	No	No	No
Urinary Incontinence	Yes	Yes	No	Yes	No	No	Yes	No	No	No	No
Leuko encephalopathy	Yes	No	Yes	Yes	Yes	Yes	No	No	Yes	Yes	No
Course	Deceased at 48 years	55 years	74 years	76 years	60 years	65 years	Deceased at 56 years	68 years	65 years	79 years	24 years
Other	Coarse facies	Kidney stones	tendinosis	Dysphagia, kidney stones	No	Fasciculation type II diabetes	Hypothyroidism, CRF	No	No	No	Hypertrichosis

Abbreviations: Pt= patient; A‐PD, atypical Parkinson's disease; CRF, chronic renal failure; HL, Hodgkin lymphoma; lymph, lymphoma; MM, multiple myomas; NEGPA, Churg–Strauss syndrome; NHL, non‐Hodgkin lymphoma; PD, Parkinson's disease; TC, thyroid carcinoma.

^a^ HGMD Professional 2023.4 accession no. NM_000153.4.

Pt1 was compound heterozygous for the p.(Ile384Thr) known pathogenetic variant [28] and the p.(Arg184Cys) disease‐associated polymorphism [29] in the *GALC* gene. *In silico* analysis demonstrated a high probability of a deleterious effect for both the p.(Arg184Cys) variant and the (p.Ile384Thr) pathogenetic variant in the *GALC* gene. The p.(Arg184Cys) variant was also detected in Pts 5–10 of the selected cohort (Table 1).

Qualitative and quantitative *GALC* mRNA analysis, performed in leukocytes from Pt1, did not reveal abnormalities, and CNV analysis did not detect *GALC* rearrangements. Whole exome sequencing performed on Pt1 and his parents ruled out known pathogenic autosomal recessive or dominant causes of monogenic inherited disorders. Pt2 harbored the heterozygous monoallelic p.(Tyr370Ser) and Pt3 the c.1034‐2A>G, both known to be disease‐causing variants [30] (Table 1). Both patients also carried additional pathogenic variants in lysosomal genes other than *GALC*, which result in LSDs with autosomal recessive inheritance. Specifically, Pt2 was a heterozygous carrier for the c.1417+5G>A known pathogenic variant in *HEXB* [31, 32], which encodes the beta subunit of the enzyme hexosaminidase that is defective in glycosphingolipidosis Sandhoff disease. Pt3 was heterozygous for the known pathogenic variant c.1874_1875del, p.(Arg625Ilefs*7) in the beta‐glucuronidase (*GUSB*) gene [33], which is altered in mucopolysaccharidosis VII.

Pt4 carried the p.(Tyr319Cys) pathogenetic variant, which was previously associated with late‐onset KD [13]. Pt11 was heterozygous for the p.(Leu238Leu) synonymous variant (Table 1). This transversion has a very low frequency in the general population and was not reported previously. According to the *in silico* analysis, there is no predicted impact on the donor/acceptor splicing site. However, the c.714C>G variant is predicted to create a new recognition motif for the serine–arginine‐rich protein 55 (SRp55) that plays significant roles in both constitutive and alternative pre‐mRNA splicing [34].

Table 2 reports the frequencies of the identified variants in the selected cohort. We compared the data from the gnomAD v4.1.0 database and the data from 1000 exome analyses on the Italian population [35]. The known pathogenic variants and the synonymous variant are absent in the general healthy Italian population, as shown in Table 2.

**TABLE 2:** Frequency of GALC identified variants in healthy population databases.

Variant	gnomAD v4.1.0 (https://gnomad.broadinstitute.org/)		NIG‐ExIT (http://nigdb.cineca.it) (Birolo et al. 2020)	dbSNP database (https://www.ncbi.nlm.nih.gov/snp/)
	Frequency	Homozygotes		
c.1151T>C (p.Ile384Thr)	12/1,613,352 (0.00074%)	no	—	rs1376496659
c.1109_1110 delACinsCG p.(Tyr370Ser)	—	—	—	—
c.714C>G, p.(Leu238Leu)	8/1,614,032 (0.00049%)	No	—	rs748497197
c.550C>T p.(Arg184Cys)	82,345/1,612,988 (5.1%)	2422	129/1552 (5 homozygotes)	rs1805078
c.956A>G p.(Tyr319Cys)	1275/1,613,458 (0.079%)	14	—	rs183105855
c.1034‐2A>G	6/1,611,810 (0.00037%)	no	—	—

*Note:* — Absent.

### 3.2 Enzyme Assays

**FIGURE 1:**
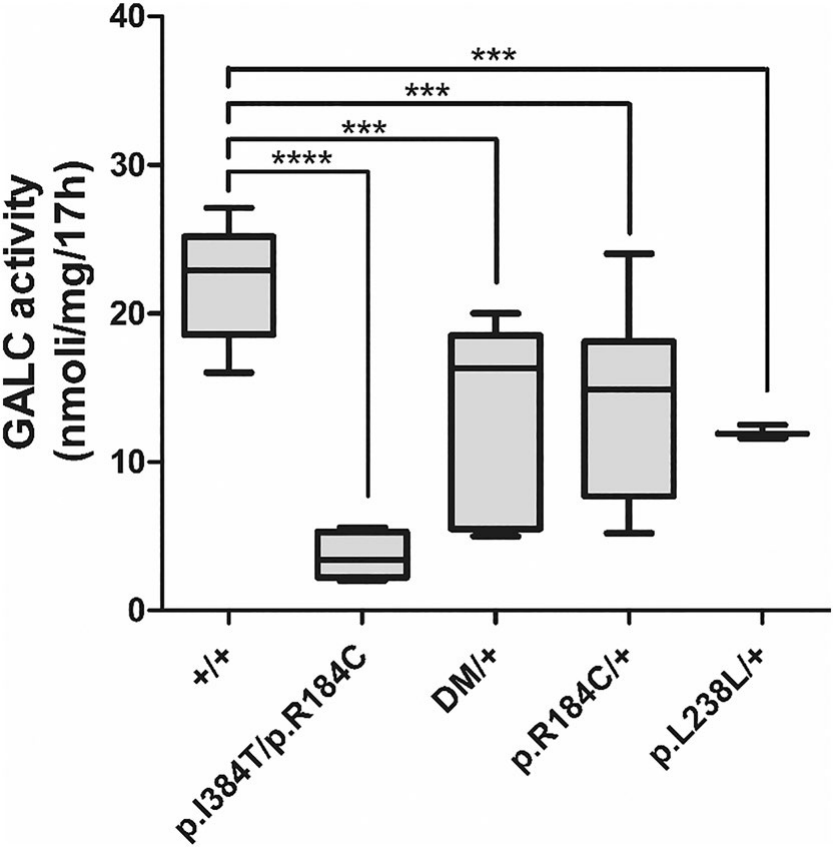
Galactosylceramidase (GALC) activity levels in patients’ leukocytes. Quantitation of GALC activity assayed in protein extracts from derived‐blood leukocytes of controls (+/+), patient with compound heterozygous status (p.I384T+p.R184C), GALC variant carrier (DM/+ = p.Y370S, c.1034‐2A>G, p.Y319C), disease‐associated polymorphism carrier c.550C>T (p.R184C/+) and patient with the rare synonymous variant c.714C>G. Box and whisker plots display the median, minimum and maximum values for each group. Borders of the box denote the 25th percentile and 75th percentile; ***p < 0.005, ****p < 0.0001.

GALC assays performed both with the fluorimetric and the LC–MS/MS methods identified a pathological decrease in enzyme levels in the sample obtained from Pt1 (Table 3, Figure 1). The LC–MS/MS method performed in the dry blood spots failed to reveal any significant difference in GALC enzyme activity in the other patients' samples.

**TABLE 3:** *GALC* genetic analysis and related enzyme assays in the patient with Krabbe disease (Pt1) and in his family members.

Patient	*GALC* ^a^ genotype	GALC^b^ activity	
		Fluorimetric assay (n.v. 18.0–75.0 nmol/mg/17 h)	LC–MS/MS assay (n.v. > 0.7 μmol/L/h)
Proband	c.1151T>C+c.550C>T; [p.Ile384Thr]+[p.Arg184Cys]	3.6	0.3
Father	c.550C>T; [p.Arg184Cys]	23.9	3.3
Mother	c.1151T>C; [p.Ile384Thr]	6.0	1.3
Daughter 1	c.1151T>C; [p.Ile384Thr]	6.8	0.7
Daughter 2	c.550C>T; [p.Arg184Cys]	10	1.6

Abbreviations: LC–MS/MS, liquid chromatography–tandem mass spectrometry; n.v., normal values.

^a^ *GALC* gene GenBank‐EMBL accession no. NM_000153.4.

^b^ UniProt accession number P54803.

Instead, the fluorimetric method showed a severe reduction of GALC enzyme activity in Pts 2–4 compared to healthy controls (Figure 1). The six patients (Pts 5–10) carrying the p.(Arg184Cys) variant and Pt11 carrying the p.(Leu238Leu) also exhibited a significant reduction of GALC activity (Table 1, Figure 1).

The relatives of Pt1, carrying the p.(Ile384Thr) pathogenetic variant at a heterozygous state, exhibited a remarkable reduction in GALC activity compared to the normal average levels, while family members heterozygous for the p.(Arg184Cys) variant exhibited a less severe reduction (Table 3).

### 3.3 GALC Structural Analysis and Interactions

**FIGURE 2:**
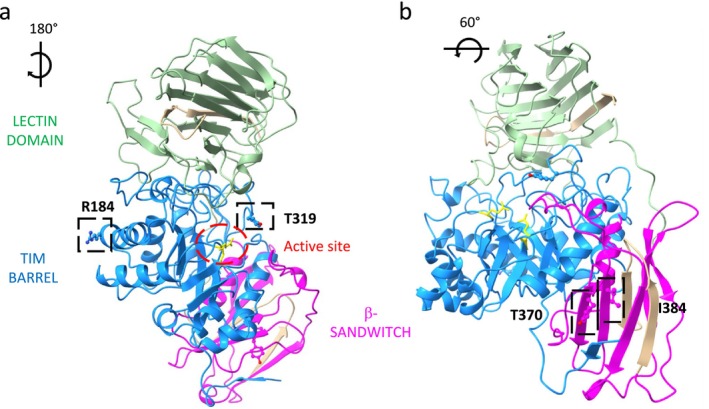
Structure of human GALC protein that comprises the 41–668 residues. (a) The residues 41–337 form TIM barrel domain (blue) in the centre of the structure, residues 338–452 β‐sandwitch domain (pink), and residues 472–668 the lectin domain (green). In detail the residues: Arg184 (R184), Tyr 319 (T319) and two residues of the active site (E198, E274) are marked in yellow. (b) Focus on the residues Tyr370 (T370) and Ile384 (I384) (black boxes) localized on the β‐sandwitch domain. The entire structure of the protein was designed using the Phyre2 tool entering the sequence (http://www.sbg.bio.ic.ac.uk/phyre2/html/page.cgi?id=index) and molecular graphic was performed with UCSF ChimeraX, developed by the Resource for Biocomputing, Visualization, and Informatics at the University of California, San Francisco, with support from National Institutes of Health R01‐GM129325 and the Office of Cyber Infrastructure and Computational Biology, National Institute of Allergy and Infectious Diseases.

We mapped the GALC missense variants identified in Pts1, 2, and 4 into the GALC tri‐dimensional structural model (Figure 2). Both the p.(Ile384Thr) and p.(Tyr370Ser) are located in the beta‐sandwich domain of the GALC (Figure 2). The p.(Tyr319Cys) pathogenic variant and the p.(Arg184Cys) disease‐associated polymorphism are located on the surface of the TIM barrel central domain comprising residues 41–337.

**FIGURE 3:**
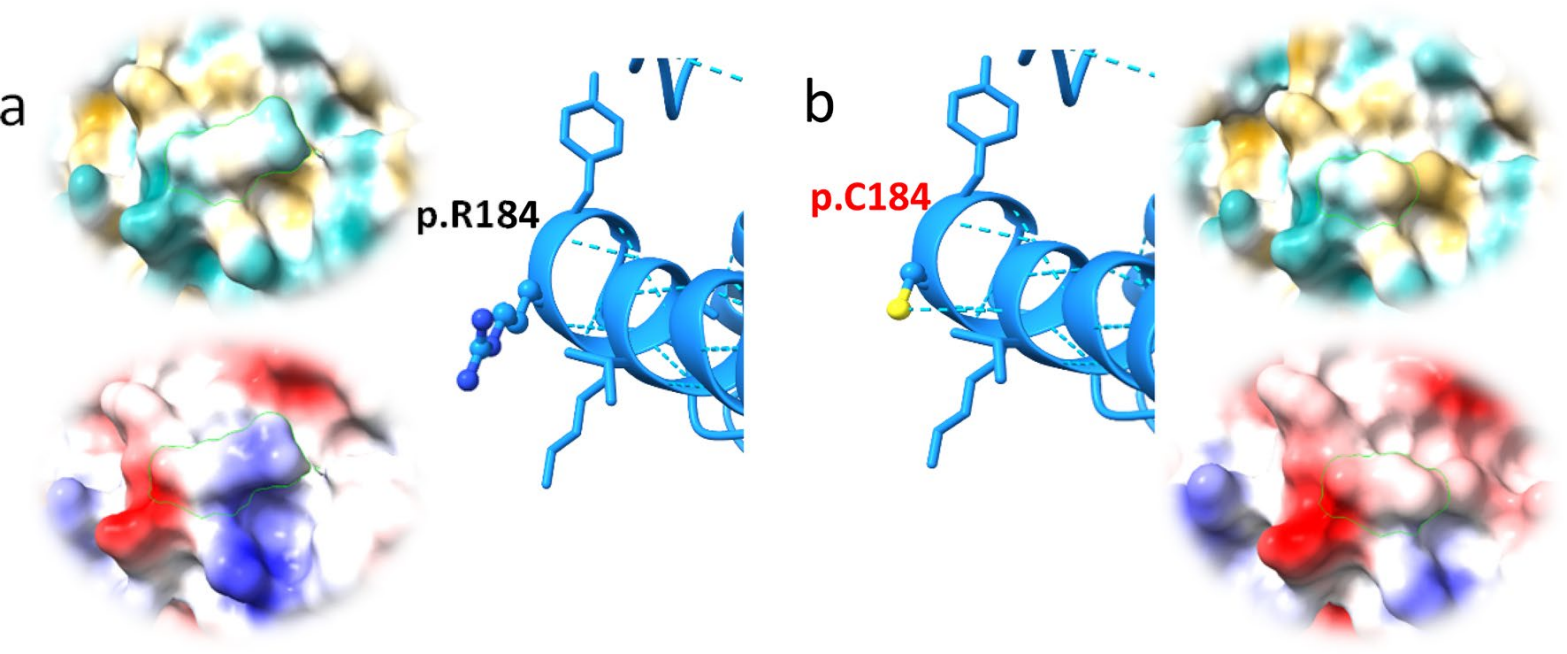
Molecular graphic of GALC performed with UCSF ChimeraX. Electrostatic (circle up) and hydrophobicity (circle down) molecular display. (a) Focus on the residue p.R184 located on α‐helix at the TIM barrel domain. Hydrophobicity score: −4.5; hydrophilicity score: 3.0, (b) mutated residue p.(R184C) observed with Rotamer library Dunbrack. Chi1 parameter: −69.5° with prevalence P: 0.81 and H‐bonds predicted: 1 with V180. Hydrophobicity score: 2.5; hydrophilicity score: −1.0.

The Arg to Cys amino acid substitution at position p.184 causes the formation of a hydrogen bond in the mutated GALC protein (Figure 3). This amino acid change significantly alters the hydrophobic and electrostatic charges on the TIM barrel surface (Figure 3).

It is possible that variants reported in Table 1, and particularly the p.(Arg184Cys), disrupt the folding or lysosomal localization of the enzyme, as described for many mutated residues [36]. Pathogenetic variants located on the surface of the TIM barrel, which only mildly affect the residual GALC activity, may disrupt the binding of GALC to an activating factor, such as saposin [36]. Charged and polar groups, hydrogen bonds, and other electrostatic interactions play an important role in protein folding and protein–ligand interactions [37, 38]. Therefore, the p.(Arg184Cys) might alter the surface of the TIM barrel domain, which could in turn perturb the folding of the enzyme or protein‐to‐protein interactions.

**FIGURE 4:**
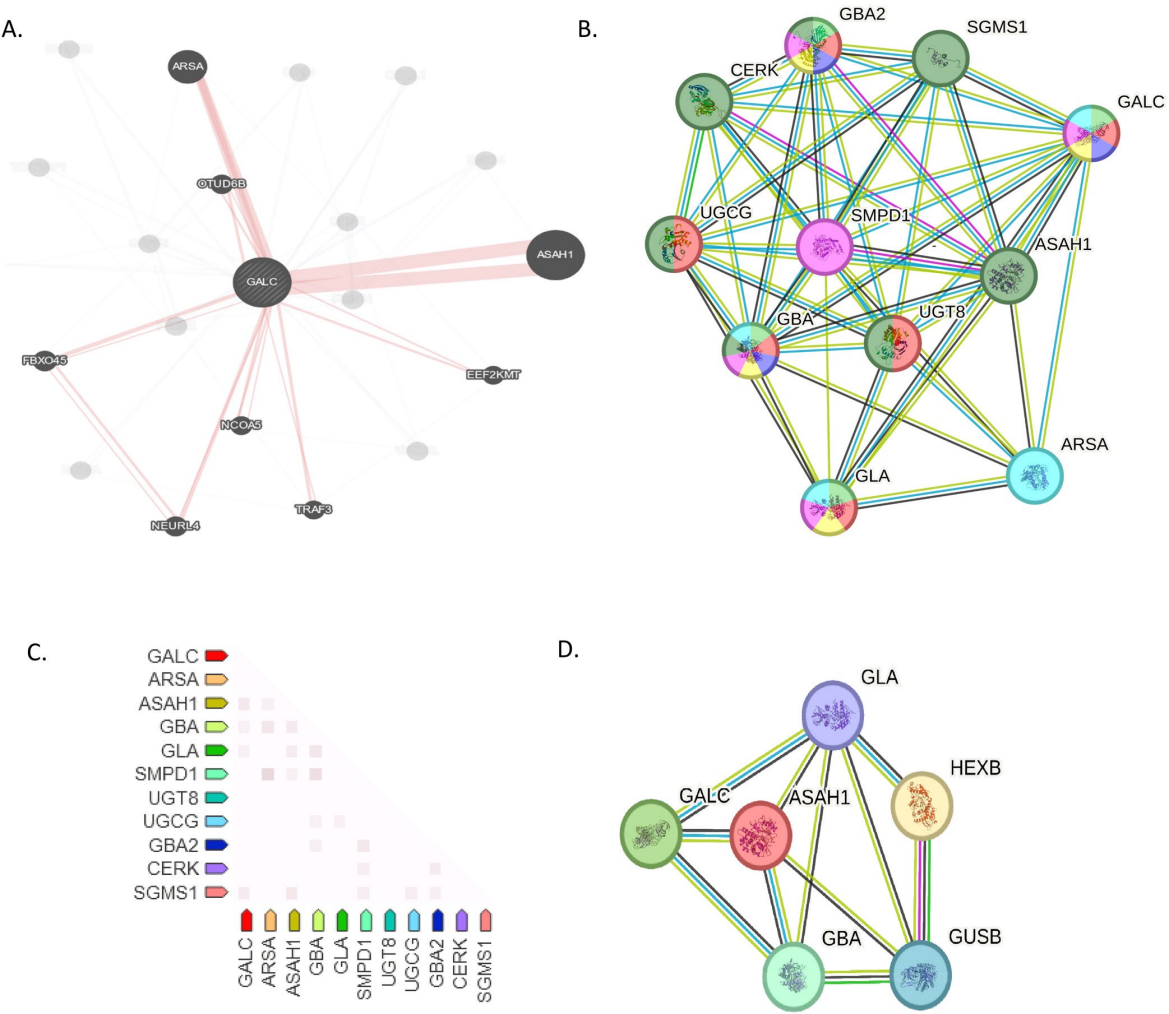
GALC networks and main functional enrichments. (A) Focus on physical interactions (https://genemania.org/); (B) Focus on biological process and molecular processes based on gene ontology and on Wikipathways (https://www.wikipathways.org/). Pink= glycosylceramide catabolic process; red = glycosylceramide metabolic process; blue = galactosylceramidase activity; green = hydrolase activity, hydrolysing *O*‐glycosyl compounds; yellow = hydrolase activity acting on glycosyl bonds; deep‐green = sphingolipid pathway; light‐blue = degradation pathway of sphingolipids, including diseases; (C) GALC Gene coexpression network. Scores are based on RNA expression patterns and on protein co‐regulation provided by ProteomeHD (https://www.proteomehd.net/proteomehd); (D) Interactions between selected proteins including GALC, GBA, GLA, ASAH1, GUSB and HEXB.

*In silico* prioritization of gene interactions showed that GALC presented physical interactions with *N*‐acyl sphingosine amidohydrolase 1 (ASAH1) and arylsulfatase A (ARSA) (Figure 4A). Based on biological processes and molecular functions, we observed a strong connection of GALC with GBA and alpha‐galactosidase (GLA) (Figure 4B). When considering functional network clusters, the selection of sphingolipid metabolism connected GALC with ASAH1, and when involving the sphingolipid catalytic process and the ceramide catabolic process in particular, GALC is connected to ARSA (Figure 4B). Coexpression data showed that GALC is mainly linked not only to ASAH1, but also to GBA and GLA (Figure 4C).

Both ceramide and sphingosine are produced by GALC, which acts on psychosine and Gal‐Cer [15]. Based on *in silico* functional association networks, the metabolic pathways of GALC are directly linked to glycosphingolipid metabolism, which involves HEXB, and indirectly linked to the glycosaminoglycan pathway. This pathway involves both HEXB and GUSB (see Figure 4D).

### 3.4 Clinical Observations

Patients' anamnesis revealed evidence of familial neurodegenerative disease in Pts1, 2, 8, 9, 10 (Table 1).

Pt1 was diagnosed with late‐onset KD. At 9 months old, an ossified and enlarged anterior fontanelle was observed, leading to systemic osteosclerosis, craniosynostosis, and intracranial hypertension. At age 2, blindness, unsteady gait, and hydrocephalus became apparent. A spinoperitoneal shunt was performed the following year to treat hydrocephalus. At age 9 years, the child manifested a first generalized seizure. From the third decade of life, the cognitive and motor functions severely declined, with memory loss, dysarthria, ataxia, apraxia, and behavioral deterioration. The patient died due to brain hemorrhage at 48 years of age.

In the selected cohort, several symptoms were recurrent, such as parkinsonism, neuropathy, and psychiatric manifestations, including depression, anxiety, and hyperactivity. Our study revealed a high prevalence of immunological features and cancer (see Table 1). Ocular manifestations leading to impaired vision ranged from cataracts to retinopathy, and total blindness was detected in the patient with KD (Pt1).

Several patients in the cohort exhibited common symptoms of KD, such as vascular leukoencephalopathy, urinary incontinence, impaired vision, and neuropathy (Table 1).

Among the three patients with known *GALC* monoallelic pathogenetic variants, Pts2 and 3 manifested sensory organ diseases, cancer, and neuropathy, in line with previous reports on late‐onset Krabbe patients. Specifically regarding neuropathy, Pt2 experienced numbness, reduced ability to sense temperature changes, and muscle cramps, while Pt3 reported burning and stabbing pain upon standing, with poor balance and coordination. Pts3 and 4 MRI revealed leukoencephalopathy, an imaging sign typical of KD and also detected in Pt1.

## 4 Discussion

Here, we report a high incidence of *GALC* gene variants in a selected cohort of patients with neurodegenerative conditions. Previous studies estimated that KD affects 1 in 100,000 to 250,000 live births [13, 14], with carrier status predicted to be around 1 in 150 to 1 in 250 live births. However, our findings indicate a much higher carrier status of KD in the selected neurodegenerative cohort, specifically about 1 in 28 (*z* score: 4.05, *p* < 0.005).

Most of the patients in our cohort exhibited immunological features ranging from an IgA deficit, detected in Pt2, to conditions such as connective tissue disorder and Churg–Strass syndrome in Pts6 and 9, respectively. Our findings also suggest that inflammation plays a role in the progression of clinical manifestations. Reports of inflammation in KD [16] and the association of heterozygous *GALC* pathogenetic variants with autoimmune diseases of the nervous system, such as MS [18], support this hypothesis.

We also point out that half of the patients in our cohort developed oncological conditions, with Pt2 manifesting two independent forms of cancer. Clinical and experimental evidence has suggested pathobiological crosstalk between LSDs and cancer [39]. These findings support previous reports suggesting a potential connection between sphingolipid metabolism, particularly GALC, and tumor progression [40]. It has been hypothesized that GALC acts as an oncosuppressor, with its downregulation being a risk factor for developing cancer [40, 41]. A direct observation of increased prevalence of melanoma has also been previously reported in carriers of a potentially pathogenic variant of *GALC* [42]. Biochemical, molecular, genetic, and phenotypic characteristics suggest that one patient in the cohort had a slowly evolving form of KD (Pt1).

In its adult manifestations, KD is underdiagnosed and often confused with other neurodegenerative diseases [4, 11]. Presenting symptoms in Pt1, such as visual loss, unsteady gait, and seizures, are consistent with previous reports in adult‐onset KD [43]. This patient also exhibited typical focal areas of high signal intensity in the white matter on MRI, consistent with leukoencephalopathy. No signs of sensorimotor polyneuropathy were apparent.

Signs of sensory and motor neuropathy were instead present in Pts2 and 3, harboring a heterozygous *GALC* pathogenetic variant. Pt3 also exhibited atypical parkinsonism before the age of 65. PD and atypical parkinsonism have been largely linked to genetic causes [44]. In particular, previous reports have indicated that *GBA* pathogenetic variants are a significant genetic risk factor for atypical PD [44]. GALC is responsible for producing both the product (ceramide) and the substrate (glucosylceramide) of GBA in the sphingolipid catabolic pathway. Functional connections between GBA and GALC are present (Figure 4), thus supporting a possible causative role of GALC in parkinsonism. A possible role of GALC in the metabolic pathways leading to parkinsonism is also suggested by the contribution of alpha‐synuclein inclusions in the pathophysiology of KD [5, 7].

Regarding the other two patients carrying known heterozygous *GALC* pathogenetic variants, Pt4 presented with parkinsonism accompanied by severe cognitive decline. Pt2 manifested visual impairment, leukoencephalopathy, and neuropathy, which are common symptoms of adult KD, but no signs of parkinsonism.

Symptoms observed in Pts2 and 4 suggest that *GALC* defects could be a contributing factor to different neurodegenerative conditions. This hypothesis is endorsed by previous studies on heterozygous carriers of pathogenetic variants in the *GALC* gene who showed an increased risk of developing different pathological conditions, including open‐angle glaucoma [45], pulmonary diseases [46], synucleinopathies [16], and MS [18]. It could be argued that slightly deficient GALC activity could lead to progressive neurodegeneration, not severe enough to be recognizable as a definite entity such as PD. As previously suggested [16], pathogenicity might result from a slowly progressive accumulation of toxic metabolites, to the extent that symptoms are only manifested later in life.

We postulate that neurodegeneration of unknown etiology should be considered in the light of the individual's genetic background and modifying factors, a hypothesis corroborated by previous literature. For instance, in PD, the role of genetics, combined with environmental and lifestyle factors, has been firmly established, with consequent improvements in patient care [44]. A particular genetic set, along with epigenetic and environmental factors, has also been reported to significantly impact LSDs [47].

Our findings demonstrated that the p.(Arg184Cys) polymorphism exhibited a notable reduction in GALC enzyme activity in the neurodegenerative patients reported here (Pts1 and 5–10, see Table 1 and Figure 1). Although this variant is found in the general population with a frequency of about 5%, it is known to cause a reduction in GALC activity and an increase in the GALC precursor in *in vitro* systems expressing it [48]. It has also been demonstrated that the in *cis* expression of the p.(Arg184Cys) variant, along with particular pathogenetic variants, reduces residual GALC activity and alters the processing of the resulting GALC precursor [48]. The p.(Arg184Cys) variant is not sufficient to cause KD when present in the homozygous state. However, this variant has been associated with ADHD in a study on a cohort of 1520 genotyped subjects (rs1805078; estimated frequency 0.058) [19, 29].

It has been proposed that genes other than GALC and/or environmental factors could contribute to the progressive neurodegeneration of later onset KD^14^. Pts2 and 3 harbored known pathogenic variants in other lysosomal genes (respectively *GUSB* and *HEXB*). The catalytic pathways associated with HEXB include glycosaminoglycan metabolism and sphingolipid metabolism, while GUSB is a hydrolase that degrades glycosaminoglycans [49]. Figure 4 illustrates the relationships between GALC and HEXB or GUSB, which are mediated by interactions with other proteins (GLA, ASAH1, and GBA). The gene configurations detected in Pts2 and 3 might represent an additional risk factor for a neurodegenerative process since they involve many components of lysosomal catalytic pathways. Numerous findings support this. Alpha‐synuclein aggregation may result from pathogenetic variants in a number of LSD genes, including *HEXB* [5], and MS may be directly caused by pathogenetic variants in the *ARSA* gene [18]. Additionally, PD has been associated with a significant number of LSD gene variations [5, 50].

In conclusion, our research suggests that characterizing *GALC* gene variants, including the effects of disease‐related polymorphisms or synonymous variants, can be helpful when approaching the diagnostic process of neurodegenerative diseases with unknown etiology and overlapping symptoms of LSDs. However, larger studies are needed to explore potential and yet unknown disease mechanisms.

Unidentified genetic and/or epigenetic factors should also be taken into account when making a differential diagnosis of disorders considered as primarily monogenic.

## Author Contributions

**Federica Feo:** investigation, methodology, writing – review and editing, project administration, data curation. **Luciana Tramacere:** data curation, visualization, writing – review and editing, resources, funding acquisition. **Silvia Ramat:** supervision, visualization, writing – review and editing, resources, funding acquisition. **Alessandra Govoni:** investigation, writing – review and editing, validation, project administration. **Luca Caremani:** methodology, formal analysis, data curation. **Giulia Grigioni:** methodology, formal analysis, visualization, project administration. **Davide Mei:** methodology, software, formal analysis, data curation. **Silvia Falliano:** investigation, methodology, data curation, visualization. **Francesca Marin:** investigation, methodology, data curation, validation, software. **Lorenzo Ferri:** methodology, formal analysis, conceptualization. **Antonella Paoli:** investigation, visualization, supervision, project administration. **Marina Rinaldi:** investigation, methodology, software. **Giancarlo la Marca:** conceptualization, validation, supervision. **Daniela Ombrone:** investigation, methodology, supervision. **Elena Procopio:** conceptualization, visualization, supervision, project administration. **Renzo Guerrini:** conceptualization, investigation, writing – review and editing, supervision. **Amelia Morrone:** funding acquisition, writing – review and editing, project administration, supervision, data curation, resources. **Anna Caciotti:** conceptualization, investigation, writing – review and editing, supervision, writing – original draft.

## Conflicts of Interest

The authors declare no conflicts of interest.

## Supporting information


**Table S1.** List of the 64 lysosomal storage disease (LSD) genes analyzed by NGS.

## Data Availability

The data that support the findings of this study are available on request from the corresponding author. The data are not publicly available due to privacy or ethical restrictions.
