# Supplementary material for: High Prevalence of GALC Gene Variants in Adults With Neurodegenerative Conditions
Source: Eur J Neurol. 2025 May 20;32(5):e70206. doi: 10.1111/ene.70206 (PMC12090362; doi:10.1111/ene.70206)
Supplement: Supplementary file 1 — Table S1. List of the 64 lysosomal storage disease (LSD) genes analyzed by NGS. [file ENE-32-e70206-s001.doc]

| **Supplementary Table 1. List of the 64 Lysosomal Storage Disease (LSD) genes analysed by NGS.** | | | |
| --- | --- | --- | --- |
| **Gene Symbol** | **RefSeq** | **Location** | **Disease** |
| ***AGA*** | NM_000027.4 | 4q32-q33 | Aspartylglucosaminuria |
| ***AP3B1*** | NM_003664.5 | 5q14.1 | Hermansky-Pudlak syndrome 2 |
| ***AP5Z1*** | NM_014855.3 | 7p22.2 | Spastic paraplegia 48, autosomal recessive |
| ***ARSA*** | NM_000487.6 | 22q13.31-qter | Metachromatic leucodistrophy |
| ***ARSB*** | NM_000046.5 | 5q11-q13 | MPS VI |
| ***ASAH1*** | NM_177924.5 | 8p22-p21.3 | Farber |
| ***ATP13A2*** | NM_022089.4 | 1p36 | NCL 12; Kufor-Rakeb syndrome |
| ***BLOC1S3*** | NM_212550.5 | 19q13.32 | Hermansky-Pudlak syndrome 8 |
| ***CLN3*** | NM_001042432.2 | 16p12.1 | NCL 3; Batten disease; Spielmeyer-Vogt disease |
| ***CLN5*** | NM_006493.4 | 13q21.1-q32 | NCL 5 |
| ***CLN6*** | NM_017882.3 | 15q23 | NCL 6; Kufs type, adult onset |
| ***CLN8*** | NM_018941.4 | 8p23 | NCL 8; Northern epilepsy variant |
| ***CTNS*** | NM_004937.3 | 17p13 | Cystinosis |
| ***CTSA*** | NM_000308.4 | 20q13.1 | Galactosialidosis |
| ***CTSD*** | NM_001909.5 | 11p15.5 | NCL 10 |
| ***CTSF*** | NM_003793.4 | 11q13 | NCL 13; Kufs type |
| ***CTSK*** | NM_000396.4 | 1q21 | Pycnodysostosis |
| ***DNAJC5*** | NM_025219.3 | 20q13.33 | NCL 4; Parry type |
| ***DTNBP1*** | NM_032122.5 | 6p22.3 | Hermansky-Pudlak syndrome 7 |
| ***FUCA1*** | NM_000147.5 | 1p34 | Fucosidosis |
| ***GAA*** | NM_000152.5 | 17q25.2-q25.3 | Glycogenosis type II/Pompe |
| ***GALC*** | NM_000153.4 | 14q31 | Krabbe |
| ***GALNS*** | NM_000512.5 | 16q24.3 | MPS IVA |
| ***GBA*** | NM_001005741.3 | 1q21 | Gaucher |
| ***GLA*** | NM_000169.3 | Xq22 | Fabry |
| ***GLB1*** | NM_000404.4 | 3p21.33 | MPS IVB/Gangliosidosis GM1 |
| ***GM2A*** | NM_000405.5 | 5q31.3-q33.1 | Gangliosidosis GM2, activator defect |
| ***GNE*** | NM_001128227.3 | 9p13.3 | Sialuria |
| ***GNPTAB*** | NM_024312.5 | 12q23.2 | Mucolipidosis II alfa/beta, III alfa/beta |
| ***GNPTG*** | NM_032520.5 | 16p13.3 | Mucolipidosis III gamma |
| ***GNS*** | NM_002076.4 | 12q14 | MPS III D (San Filippo D) |
| ***GRN*** | NM_002087.4 | 17q21.32 | NCL 11; Aphasia, primary progressive;rontotemporal lobar degeneration with ubiquitin-positive inclusions |
| ***GUSB*** | NM_000181.4 | 7q21.11 | MPS VII |
| ***HEXA*** | NM_000520.6 | 15q23-q24 | Gangliosidosis GM2, Tay Sachs |
| ***HEXB*** | NM_000521.4 | 5q13 | Gangliosidosis GM2, Sandhoff |
| ***HGSNAT*** | NM_152419.3 | 8p11.1 | MPS III C (San Filippo C) |
| ***HPS1*** | NM_000195.5 | 10q23.1-q23.3 | Hermansky-Pudlak syndrome 1 |
| ***HPS3*** | NM_032383.5 | 3q24 | Hermansky-Pudlak syndrome 3 |
| ***HPS4*** | NM_022081.6 | 22cen-q12.3 | Hermansky-Pudlak syndrome 4 |
| ***HPS5*** | NM_181507.2 | 11p14 | Hermansky-Pudlak syndrome 5 |
| ***HPS6*** | NM_024747.6 | 10q24.32 | Hermansky-Pudlak syndrome 6 |
| ***HYAL1*** | NM_153281.2 | 3p21.3-p21.2 | MPS IX |
| ***IDS*** | NM_000202.8 | Xq28 | MPS II |
| ***IDUA*** | NM_000203.5 | 4p16.3 | MPS I |
| ***KCTD7*** | NM_153033.5 | 7q11.21 | NCL 14; Epilepsy, progressive myoclonic 3, with or without intracellular inclusions |
| ***LAMP2*** | NM_002294.3 | Xq24 | Danon |
| ***LIPA*** | NM_000235.4 | 10q23.2-q23.3 | Wolman |
| ***MAN2B1*** | NM_000528.4 | 19cen-q13.1 | alfa-mannosidosis |
| ***MANBA*** | NM_005908.4 | 4q22-q25 | beta-mannosidosis |
| ***MCOLN1*** | NM_020533.3 | 19p13.3-p13.2 | Mucolipidosis IV |
| ***MFSD8*** | NM_152778.4 | 4q28.1-q28.2 | NCL 7; Macular dystrophy with central cone involvement |
| ***NAGA*** | NM_000262.3 | 22q11 | Schindler |
| ***NAGLU*** | NM_000263.4 | 17q21 | MPS III B (San Filippo B) |
| ***NEU1*** | NM_000434.4 | 6p21.3 | Sialidosis |
| ***NPC1*** | NM_000271.5 | 18q11-q12 | Niemann-Pick type I |
| ***NPC2*** | NM_006432.5 | 14q24.3 | Niemann-Pick type II |
| ***PPT1*** | NM_000310.4 | 1p32 | NCL 1 |
| ***PSAP*** | NM_002778.4 | 10q21-q22 | Metachromatic leucodistrophy, Krabbe, Gaucher |
| ***SCARB2*** | NM_005506.4 | 4q21.1 | epilepsy, progressive myoclonic 4, with or without renal failure and unverricht-lundborg syndrome |
| ***SGSH*** | NM_000199.5 | 17q25.3 | MPS III A (San Filippo A) |
| ***SLC17A5*** | NM_012434.5 | 6q14-q15 | Sialic acid storage disease; Salla disease |
| ***SMPD1*** | NM_000543.5 | 11p15.4-p15.1 | Niemann-Pick |
| ***SUMF1*** | NM_182760.4 | 3p26.2 | Multiple sulfatase deficiency |
| ***VPS33A*** | NM_022916.6 | 12q24.31 | Mucopolysaccharidosis-plus syndrome |
| ***TPP1*** | NM_000391.4 | 11p15 | NCL 2; Spinocerebellar ataxia, autosomal recessive 7 |
